# Sex-Dependent Alterations in Social Behaviour and Cortical Synaptic Activity Coincide at Different Ages in a Model of Alzheimer’s Disease

**DOI:** 10.1371/journal.pone.0046111

**Published:** 2012-09-24

**Authors:** Cyril Bories, Matthieu J. Guitton, Carl Julien, Cyntia Tremblay, Milène Vandal, Meriem Msaid, Yves De Koninck, Frédéric Calon

**Affiliations:** 1 Faculty of Medicine Laval University, Quebec City, Quebec, Canada; 2 Faculty of Pharmacy, Laval University, Quebec City, Quebec, Canada; 3 Centre Hospitalier de l’Université Laval (CHUL) Research Center, Quebec City, Quebec, Canada; 4 Quebec Mental Health Institute, Quebec City, Quebec, Canada; University of Florida, United States of America

## Abstract

Besides memory deficits, Alzheimer’s disease (AD) patients suffer from neuropsychiatric symptoms, including alterations in social interactions, which are subject of a growing number of investigations in transgenic models of AD. Yet the biological mechanisms underlying these behavioural alterations are poorly understood. Here, a social interaction paradigm was used to assess social dysfunction in the triple-transgenic mouse model of AD (3xTg-AD). We observed that transgenic mice displayed dimorphic behavioural abnormalities at different ages. Social disinhibition was observed in 18 months old 3xTg-AD males compared to age and sex-matched control mice. In 3xTg-AD females, social disinhibition was present at 12 months followed by reduced social interactions at 18 months. These dimorphic behavioural alterations were not associated with alterations in AD neuropathological markers such as Aβ or tau levels in the frontal cortex. However, patch-clamp recordings revealed that enhanced social interactions coincided temporally with an increase in both excitatory and inhibitory basal synaptic inputs to layer 2–3 pyramidal neurons in the prefrontal cortex. These findings uncover a novel pattern of occurrence of psychiatric-like symptoms between sexes in an AD model. Our results also reveal that functional alterations in synapse activity appear as a potentially significant substrate underlying behavioural correlates of AD.

## Introduction

Alzheimer’s Disease (AD) is the most common neurodegenerative disorder and the first cause of dementia in the elderly [1]. Besides memory deficits, the most widely identified and studied symptoms of Alzheimer’s disease (AD), a number of less well characterized but frequent behavioural symptoms of dementia, including social disinhibition, apathy, anxiety, agitation, and irritability are part of the clinical expression of AD [2], [3]. Among them, insidious alterations of personality and social interactions are particularly distressful. Unfortunately, these neuropsychiatric symptoms are very difficult to treat, often placing a heavy burden on both patients and caregivers [2], [4].

For almost two decades, transgenic animals overexpressing human mutant amyloid-β (Aβ) precursor protein (APP) and/or presenilin have been extensively used as models to induce and study Aβ deposition and memory loss as seen in AD [5], [6]. Among them, a triple-transgenic model (3xTg-AD), displaying Aβ plaques, tau-laden neurofibrillary tangles and age-dependent alterations in memory function, was developed to mimic more closely AD neuropathology [7]–[10]. Despite a growing number of studies on neuropsychiatric symptoms associated with AD such as aggressive behaviour and anxiety (for reviews [10], [11]) changes in social behaviour remain relatively overlooked in transgenic models compared to studies on memory or other cognitive and non-cognitive deficits [12]–[15]. Most importantly none of these studies clearly associated changes in social behaviour with specific neurobiological changes.

To fill this gap, we investigated here the evolution of social behaviour during the disease progression in the 3xTg-AD mouse. We found biphasic alterations in social behaviour in 3xTg-AD mice at different ages for males and females. Although the expression of typical molecular correlates of AD, such as Aβ or tau, increased with age, their association with social dysfunctions differed according to the sex of the animals. Yet, by recording the synaptic activity aiming at layer II/III pyramidal cells in the medial prefrontal cortex (mPFC), a region known to be critical for mediating depression and social behaviour [16]–[19], we found that changes in basal synaptic activity coincided with the sex and age-dependent behavioural alterations observed in 3xTg-AD mice. Thus, our results suggest that changes in synaptic activity in the mPFC may underlie social behaviour alterations in AD.

## Materials and Methods

### Animals

#### Ethics statement

The use of animals was approved by the Laval university animal ethics committee (approval ID = 07−113 and 07–061) and all procedures were performed according to the guidelines of the Canadian Council on Animal Care. Only healthy animals without any evidence of tumors or disease were used to generate behaviour, electrophysiological or biochemical endpoints included in the present study.

Animals were produced and maintained in the animal facilities of the CHUL Research Center at 22±1°C under a 12 h light/dark cycles regime. The 3xTg-AD mouse line was produced from the cointegration of the APP and tau transgenes in the same genetic locus, into single-cell embryos from homozygous PS1-knockin mice, generating mice with the same genetic background. Non-transgenic (NonTg) mice used here are derived from littermates of the original PS1-knockin mice and are on the same background as homozygous 3xTg-AD mice (C57BL6/129SvJ) [9]. Water and food were available ad libitum. NonTg and 3xTg-AD mice were divided in 4 groups according to sex and age, for a total of 8 groups of at least 8 animals. As group-housing has a limited impact on the behaviour of 3xTg-AD mice [12], 2–5 animals were housed in unisexual groups in each polycarbonate standard cages (40 cm×22 cm×18 cm). Behavioural testing and electrophysiological experiments were performed at the age of 12 (middle-aged) or 18 month (aged).

### Social Interaction Paradigm

Animals underwent a social interaction test following a previously established protocol [20]–[22]. To reduced the number of aggressive behaviours and facilitate social interactions, all tests were performed in empty standard cages (40 cm×22 cm×18 cm) [23]. Pairs of age-matched animals, unfamiliar with each other, were placed in the unfamiliar test arena for an observation period of 20 min. To focus on the effects of the transgenes, each experimental couple (dyad) consisted of a 3xTg-AD mouse paired with a sex- and age-matched NonTg mouse from the same genetic background. Within dyads, NonTg and 3xTg-AD animals were age-matched to abolish the confounding factor of age effects on behaviour. Furthermore, couples of sex-matched animals were used to avoid contamination by sexual behaviours in social interaction analysis. Thus, the response of each animal was analysed with respect to that of the sex and age-matched partner in the same observation episode (i.e. within the same dyad, in the same experimental context). A single session per dyad was performed to avoid biases stemming from the development of repeated tests-induced social hierarchy, social recognition or social stress, which are known to induce major behavioural and physiological changes as observed in social defeat model of depression [17], [24], [25].

Because it is the relative change in social interaction between the two partners within each dyad that is relevant to investigate the effect of the genotype at each age and for each sex, data were normalized to the corresponding NonTg partner values (i.e., each dyad serves as its own control; raw values are provided in Table S1).

As our interest was primarily to measure the initiation of active social behaviour sequences (i.e. the tendency of the subject mouse to approach another mouse and engage in social interactions), social interaction events were recorded when a social interaction was initiated, i.e. beginning with contact of the test partner and continuing until one of the two animals disengaged from the exchange. Disengagement was defined as when an animal turns away or leaves the proximity of its partner. [26]–[29]. Behaviours were documented as social events (sniffing, following, grooming the partner, crawling over or under), aggressive events (wrestling or biting), number of conspecific-following behaviour, number of conspecific-escapes, and number of grooming. Finally, a global score of total social interaction events was established for each mouse and then averaged for each group. After each trial, animals were returned to their home cage and the arena was changed to a new and clean one, to avoid any odor cues from one pair to the next one.

### Open Field Paradigm

Locomotor activity was evaluated using an open field system (San Diego) Instruments, CA) consisting of 10 Plexiglas chambers (40 cm×40 cm) during a one hour session. Horizontal voluntary fine and ambulatory movements were detected using a photobeam activity system [30].

### Electrophysiology

#### Subjects

Electrophysiological data were obtained from a total of thirty six naive mice, 18 3xTg-AD and 18 NonTg mice divided into 4 groups according to sex and age.

*Slice preparation and electrophysiology*: Mice were deeply anaesthetized with ketamine and xylazine and decapitated. The brain was removed quickly and placed in an ice-cold solution containing (in mM) 210 sucrose, 3.0 KCl, 0.75 CaCl_2_, 3.0 MgSO_4_, 1.0 NaH_2_PO_4_, 26 NaHCO_3_, and 10 glucose, saturated with 95% O_2_ and 5% CO_2_. Coronal slices of the frontal cortex including the prelimbic area were 250 µm thick and kept in artificial cerebral spinal fluid (ACSF) containing (in mM) 124 NaCl, 3.0 KCl, 1.5 CaCl_2_, 1.3 MgSO_4_, 1.0 NaH_2_PO_4_, 26 NaHCO_3_, and 20 glucose, gassed with 95% O_2_−5% CO_2_ at room temperature. Slices were allowed to recover for at least 1 h before recording. A slice was then transferred to a chamber exposed to ACSF and flowing at a rate of 2–3 ml/min. Recordings were performed between 32–34°C.

#### Whole-cell patch-clamp recording

Patch pipettes (6–8 MΩ) were pulled from borosilicate glass capillaries (World Precision Instruments) and filled with an intracellular solution (pH 7.2; 275–280 mOsm) composed of (in mM): 100 Cs gluconate, 5 CsCl, 10 HEPES, 2 MgCl_2_, 1 CaCl_2_, 11 BAPTA, 4 ATP, 0.4 GTP, and 0.25% Neurobiotin (Vector Laboratories, Burlingame, CA). The junction potential of the pipette was corrected by subtracting 11.4 mV from recorded membrane voltages. A Multiclamp 700B amplifier (Axon Instruments, Foster City, CA) was used for the recording. The access resistance was monitored throughout each experiment and only recordings with stable access were used. Access resistance was not compensated. At the beginning of each recording, 200-ms-long, hyperpolarizing pulses were used to measure the input resistance of each neuron. Experiments were conducted using the Clampex program (pClamp 9.2, Axon Instruments); data were collected without filter and digitized at 10 kHz. The extracellular potassium concentration was raised to 5 mM to increase the frequency of PSCs. Immediately after breakthrough, Tetrodotoxin (TTX; 1 mM from RBI) was added to the ACSF to block voltage-gated sodium channels and isolate action potential-independent miniature post synaptic currents (mPSC). Whole-cell patch-clamp recordings were performed in voltage-clamp mode while maintaining the membrane potential either at the reversal potential for GABA_A_ receptor-mediated PSCs (−60 mV) to isolate mEPSCs or at the reversal potential for ionotropic glutamate receptor mediated mEPSCs (0 mV) to isolate mIPSCs. This experimental strategy allowed us to record mEPSC and mIPSC activity from the same cell. As such data were obtained from a total of 65 cells, mEPSC means were obtained from 54 cells (mean 6.9 cells per group) and mIPSC means were obtained from 48 cells (mean 6 cells per group). Electrophysiological recordings were performed on naïve animals to preclude effects of behavioural tests on synaptic activity in medial prefrontal cortex [17], [19], [31]. To avoid any long term effect of the application of TTX to the whole bath, only one cell per slice was recorded.

#### Data analysis

Data were filtered at 1 kHz. The Clampfit 9.2 (molecular device) and Origin 8.0 (OriginLab, Northampton, MA) software were used to perform analyses.

### ELISA and Western Immunoblotting

To collect molecular endpoints, animals were perfused with 1X phosphate buffered saline (PBS) containing a cocktail of protease inhibitors (SIGMA*FAST*™, Sigma–Aldrich, St. Louis, MO) along with phosphatase inhibitors (50 mM sodium fluoride and 1 mM sodium pyrophosphate). Frozen extracts of the frontal cortex were dissected and kept at −80°C. Homogenates from cytosol (TBS-soluble), membrane (detergent-soluble) and detergent-insoluble (formic acid–soluble) fractions were generated for ELISAs and Western immunoblotting analyses as described [32], [33]. More specifically, total tau and hyperphosphorylated tau in soluble and insoluble fractions were assessed as shown previously [31], [32]. Insoluble and soluble Aβ_40_ and Aβ_42_ were measured using High Sensitive Human β-Amyloid (1–42) and (1–40) ELISA kit (WAKO, Osaka, Japan) as described [34]. Protein concentrations in samples were determined using bicinchoninic acid assays (Pierce, Rockford, IL) and equal amounts of protein per sample (15 µg of total protein per lane) were added to Laemmli’s loading buffer, heated to 95°C for 5 min before loading, and subjected to sodium dodecyl sulfate-polyacrylamide gel electrophoresis. Proteins were electroblotted onto PVDF membranes (Millipore, MA) before blocking in 5% nonfat dry milk and 1% bovine serum albumin (BSA) in PBS containing 0.1% of polysorbate-20 for 1 h. Membranes were immunoblotted with appropriate primary and secondary antibodies followed by chemiluminescence reagents (KPL, Gaithersburg, MD). Band intensities were quantified using a KODAK Image Station 4000 MM (Molecular Imaging Software version 4.0.5f7, KODAK, New Haven, CT). The following primary antibodies were used in Western immunoblotting experiments: anti-drebrin, clone MX823 (Progen, Heidelberg, Germany), anti-PSD-95 (Upstate Biotech, Lake Placid, NY), anti-synaptosome-associated protein-25 (SNAP-25) (Sternberg Monoclonals, Lutherville, MD), anti-synaptophysin (Chemicon international, Temecula, USA), anti-actin (ABM, Richmond, BC, Canada), anti-NeuN (Chemicon), anti-total tau, clone tau-13 (Covance, Berkeley, USA), anti-phospho tau, clone CP13 (gift from Dr Peter Davies, Albert Einstein College of Medicine, New York, USA), clone AT270 (Pierce), clone AD2 (Bio-Rad, Herculus, CA), anti-APP/Aβ, clone 6E10 (Chemicon).

### Statistical Analysis

All results are expressed as means +/− SEM. Statistical analysis of behavioural performance was conducted using Mann-Whitney non-parametric U tests. Statistical analyses of biochemical measurements were performed either by ANOVA (equal variance) followed by Tukey-Kramer (unequal sample sizes) or Newman-Keuls (equal sample sizes) post-hoc tests or by Welch’s ANOVA (unequal variance) followed by a Dunnett’s post-hoc test. In addition, logarithmic transformations were applied to reduce variance and deviations from normal distributions, when needed. Two- or three-way ANOVA were used to study the effect of genotype, sex and age. Correlations were performed using linear regression to generate Pearson product moment correlation coefficients.

All statistical analyses were conducted using the JMP Statistical Analysis Software (version 5.0.1). For electrophysiological data, three-way analyses of variance were conducted to study the potential interaction of genotype, sex and age on synaptic activity. Finally, planned comparison tests were performed with Student t-test for orthogonal comparison of genotype effect at different ages for each sex in Origin 8.0 software (OriginLab). Data were considered statistically significant at *p*<0.05.

## Results

### Sex–dependent Behavioural Abnormalities can be Observed at Different Ages in the 3xTg-AD Mice

**Figure 1:**
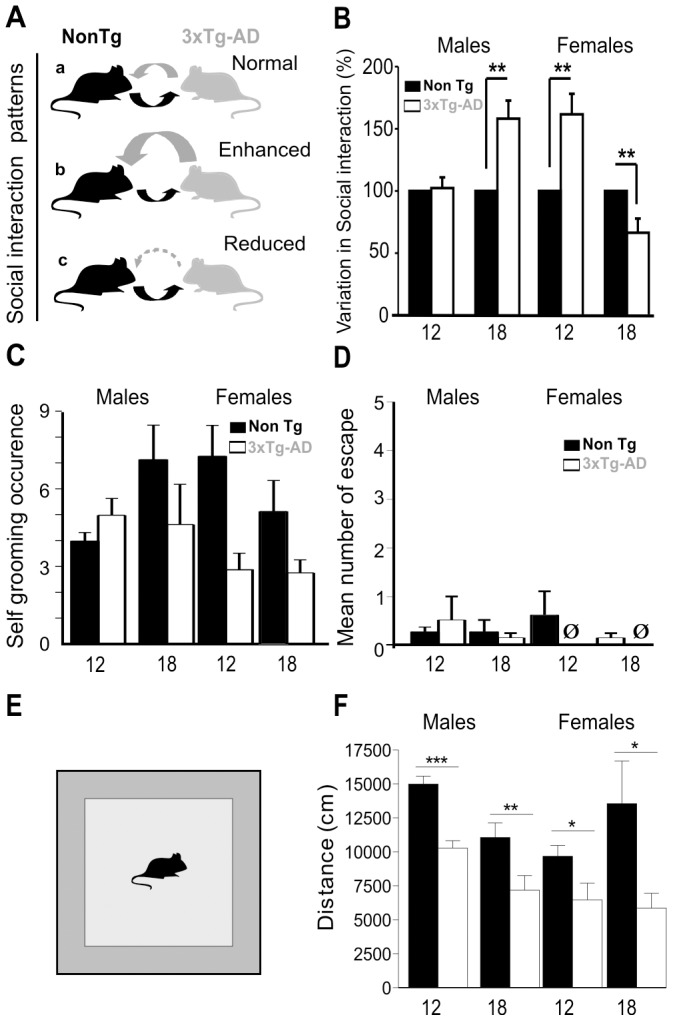
Sex-dependent behavioural abnormalities emerge at different ages in 3xTg-AD mice. (**A**) Behavioural paradigm used to study social interactions in 12-month-old and 18-month-old male and female 3xTg-AD mice, along with age- and sex-paired non-transgenic controls (NonTg). Based on the number of social events recorded during experiments, 3 different patterns of social interaction were observed, as depicted from (**a**) to (**c**). (**B**) A marked increase in social interactions was seen in 3xTg-AD females as early as 12 months of age, when compared to NonTg controls, while no difference was observed in males. However, 3xTg-AD males displayed such a social disinhibition after reaching 18 months of age when compared to age- and sex-matched NonTg animals. In contrast, the social behaviour of 18-month-old females was markedly reduced. Social events were expressed as means ± SEM. **p<0.01, *n* = 8 per group. No difference in self grooming (**C**) or escape behaviour (**D**) were observed. (**E and F**) 3xTg-AD mice travelled less in an open field task. Ø indicates that animals of the group did not demonstrate this behaviour.

As social behaviour impairment is frequent in AD, we investigated the social behaviour of 3xTg-AD mice at different ages. Given that gender differences in incidence, time of onset, and/or degree of severity have been described for many neuropsychiatric diseases, including AD [35], [36], and can also be observed in mouse models [35], [37], sexual differences were taken into account in our investigations. We thus designed a social interaction paradigm to evaluate the consequence of the genetically-induced Aβ and tau pathologies on the initiation of social interaction. To this end, we quantified the number of social events, such as sniffing, crawling over or under, following, escape behaviour, and grooming the partner, (Fig. 1 *A*) between a 3xTg-AD mouse and its age- and sex-matched Non transgenic (NonTg) mouse (derived from the same genetic background, see Material and Methods section for details).

At 12 months of age (middle-aged) the number of social events was similar between 3xTg-AD and NonTg males (Fig. 1 *B*). However, at 18 months of age (aged mice), a drastic and specific increase in the number of social events was displayed by 3xTg-AD males (p<0.01; Fig. 1 *B* and Table S1). Interestingly, such an increase in the number of social events was already present at 12 months of age for female 3xTg-AD mice (p<0.01, Fig. 1 *B* and Table S1) compared to their sex- and aged-matched NonTg dyad partners, indicating that changes in pattern of social behaviour occurred earlier in females than in males (Fig. 1 *B*). In contrast, 18-month-old 3xTg-AD females displayed a significant reduction in the initiation of social contacts compared to their NonTg partners (p<0.01; Fig. 1 *B* and Table S1). Thus the disinhibition observed in 12 months old females, i.e. the increase in the total number of investigations, was no longer evidenced in older animals, suggesting that early social memory deficits cannot fully explain the disinhibition observed.

This approach, i.e. testing only with 3xTg-AD *vs.* NonTg pairs of aged- and sex-matched animals, minimized the occurrence of aggressive behaviours and anxiogenic situations thus allowing us to focus on differences in social interactions directly linked to the AD-related transgenes within each sex and age group, with limited environmental confounds. Consistent with this, we did not observe aggressive behaviours (biting, wrestling, Table S1) or changes in anxiety (self grooming or escape, Fig. 1*C and D*). Finally, enhanced social interactions may not be solely explained by locomotor hyperactivity as 3xTg-AD mice travelled less in an open field task (Fig. 1*E and F*). Taken together, our results show that 3xTg-AD mice present biphasic alterations in social behaviour, which appear at different ages in males and females.

### Changes in Aβ, Tau, or Synaptic and Neuronal Markers in the Cortex of 3xTg-AD Mice cannot Fully Explain Behavioural Alterations

To uncover the molecular substrates and mechanisms associated with the observed biphasic behavioural alterations, we examined the evolution of the key AD neuropathological markers Aβ and tau in the frontal cortex, a region considered to be critical for social behaviour [17], [38]. One of the strengths of the 3xTg-AD model is to offer the possibility to study, in the same animal, both Aβ and tau pathologies, which are significantly associated with the clinical expression of the disease, particularly when converted to their insoluble forms [33], [39]–[41].

**Figure 2:**
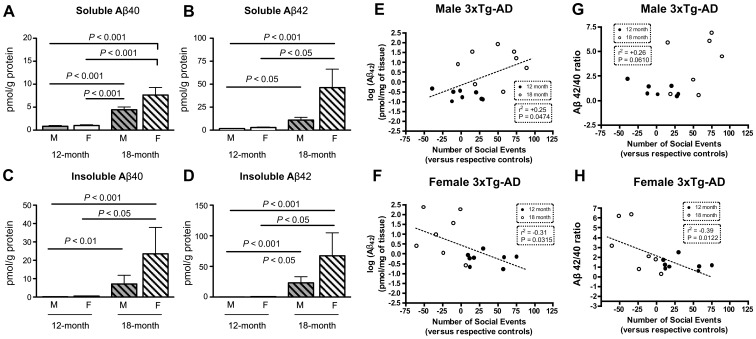
Age-dependent accumulation of Aβ peptides in soluble and insoluble protein fractions in 3xTg-AD mice. (**A–D**) ELISA (Aβ) measurements showing Aβ_40_, Aβ_42_ accumulation with age as quantified in the TBS-soluble (soluble proteins) and the formic acid-soluble (insoluble proteins) fractions from the frontal cortex of 3xTg-AD mice. Consistent trends toward increases were seen in female 3xTg-AD mice compared to males but did not reach statistical significance. Values are expressed as means ± SEM (n = 8 per group). (**E–H**) To evaluate the relation between Aβ load and social behaviour, the number of social events displayed by 3xTg-AD mice (relative to its NonTg partner) was plotted against the Aβ42 concentrations or Aβ42/40 ratio in the frontal cortex. A significant correlation could be established across the 2 groups of age (dotted lines).

**Figure 3:**
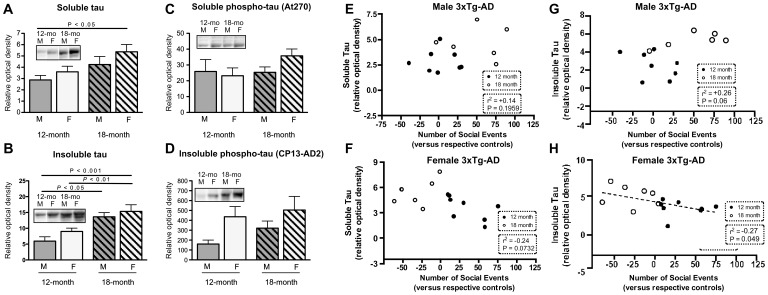
Age-dependent accumulation of tau protein in soluble and insoluble protein fractions in 3xTg-AD mice. Western immunoblots showing tau accumulation with age as quantified in the TBS-soluble (soluble proteins) and the formic acid-soluble (insoluble proteins) fractions from the frontal cortex of 3xTg-AD mice. Consistent trends toward increases were seen in female 3xTg-AD mice. (**E–H**) To evaluate the relation between tau load and social behaviour, the number of social events displayed by 3xTg-AD mice (relative to its NonTg partner) was plotted against soluble tau concentrations in the frontal cortex. A significant correlation could only be established between insoluble tau concentrations and social events in female across the 2 groups of age (dotted lines).

Consistent with Previous Studies on APP Transgenic Models [7], [42], We Found a Significant Increase in Soluble and Insoluble Aβ_40_ and Aβ_42_ Concentrations during the Aging Process (Fig. 2 *A*, B, C, *D*). Although consistent trends toward higher Aβ pathology in females were present, differences between age-matched 3xTg-AD males reached statistical significance only with two-way ANOVA for concentrations of Aβ40 in soluble fractions. Consistently, a significant relationship was established between increased concentrations of Aβ_42_ and the numbers of social events in male 3xTg-AD mice (Figure 2E). In contrast, an inverse association was observed in females (Fig. 2 *F*). As for Aβ, we observed an increase in both soluble and insoluble tau concentrations with age (Fig. 3 *A*, B, C, *D*). Tau and phospho-tau concentrations in the formic acid extracts were significantly higher in females as evidenced using two-way ANOVAs (Fig. 3 *A*, B, C, *D*) Correlative analyses also suggested that the behavioural expression of progressing tau pathology in 3xTg-AD mice differed between males and females (Fig. 3 *E, F, G, H*). These data indicate that while Aβ and tau pathologies increase in both males and females with age, their translation into social behaviours dramatically diverged between both sexes, suggesting the presence of downstream mechanisms.

We then investigated whether changes in synaptic and neuronal markers may underlie sex-dependent behavioural discrepancies observed in 3xTg-AD animals. The expression of synaptic markers such as synaptophysin and PSD-95 is known to decrease in AD [34], [43]. In our case, the concentrations of synaptophysin and PSD-95 as well as of the neuronal marker NeuN were also reduced in older 3xTg-AD animals (Table 1). However, none of these age-dependent biochemical changes were temporally associated with the sequential behavioural alterations reported above, suggesting that the dimorphism in behavioural manifestation of the disease may also lay downstream from changes in these synaptic proteins.

**Table 1:** Age and sex dependent variation in synaptic and neuronal proteins levels in the frontal cortex of 3xTg-AD mice.

	3xTg-AD 12-mo		3xTg-AD 18-mo		Two-way ANOVA(p values)	
	Male	Female	Male	Female	Sex	Age
***Synaptic markers***						
Drebrin (relative units)	29.6±8.7	24.3±3.1	19±3.6	19.7±5.8	0.699	0.198
PSD-95 (relative units)	25±2.6	20.9±1.7	18.3±1.4	17.9±1.7	0.259	0.020*
SNAP-25 (relative units)	73.1±6.4	78±5.8	63.3±4.4	65.9±6.6	0.237	0.121
Synaptophysin (relative units)	34.9±3.2	38.4±5.5	23.5±4.2	25.5±3.4	0.456	0.010*
***Neuronal Markers***						
NeuN (relative units)	49.9±2.1	49.1±2.4	41.2±2	40.8±3.8	0.814	0.001*

Notes: * p<0.05 One way ANOVA lower in older animals. All variable interactions were not significant (p>0.05).

*Abbreviations:* SNAP, synaptosome associated protein, Tg, transgenes.

Values are expressed as means ± SEM (n = 5–8 animals per group).

### Background Synaptic Activity Reflects the Dimorphic Behavioural Alterations Observed at Different Ages in 3xTg-AD Mice

**Figure 4:**
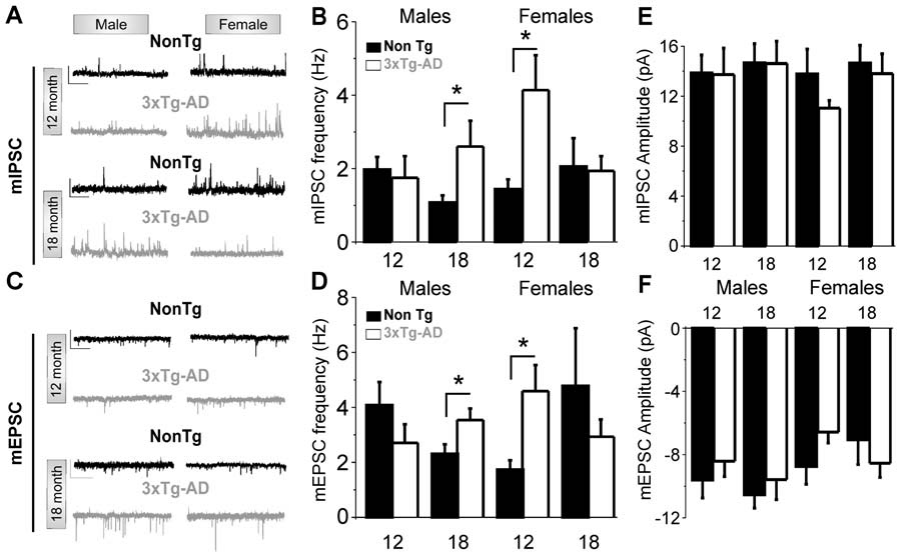
Sex-dependent synaptic hyperactivity in the prefrontal cortex is observed at different ages in 3xTg-AD mice. (**A**) Examples of miniature inhibitory post synaptic currents (mIPSCs) recorded from pyramidal cells of NonTg and 3xTg-AD mice at different ages. Scale bar 50 pA/1 s (**B**) A significant increase in mIPSC frequency was observed at 12 months in 3xTg-AD females, but was delayed until 18 months in males, *n* = 6 to 8 cells per group *p<0.05 Student t-test. (**C**) and (**D**) A significant increase in miniature excitatory post synaptic currents (mEPSC) activity was observed at 12 months in 3xTg-AD females and at 18 month in 3xTg-AD males, *n* = 5 to 7 cells per group; *p<0.05. Scale bar 25 pA/1 s. (**E and F**) No changes in mPSC amplitude were observed among the groups compared, *n* = 5 to 8 cells per group.

We then tested whether the observed behavioural alterations were associated with synaptic disorganization at the functional level. For this, we performed whole-cell recordings of miniature excitatory postsynaptic currents (mEPSC) and miniature inhibitory postsynaptic currents (mIPSC) in layer II/III pyramidal neurons in slices of the medial prefrontal cortex. Three-way analysis of variance of mEPSC and mIPSC (table S2 and S3) frequencies revealed significant interaction between age, sex and genotype, with a main effect of genotype on mIPSC frequency (with a higher mIPSC synaptic activity in 3xTg-AD cells) while no main effect could be isolated for mEPSC frequency. We next focused on the effect of genotype on synaptic activity at different ages in males and females. We revealed a significant increase in both mIPSC and mEPSC frequencies in 12-month-old 3xTg-AD females and 18-month-old 3xTg-AD males (p<0.05; Fig.4 *A*, B, C, *D*) when compared to age- and sex-matched NonTg animals. No difference in input resistance was observed between the groups (Data not shown). Since neither mIPSC nor mEPSC amplitudes were altered (Fig. 4 *E* and *F*), changes in presynaptic activity are likely to have exerted a prominent role in the genesis of the changes we observed in our study.

## Discussion

**Figure 5:**
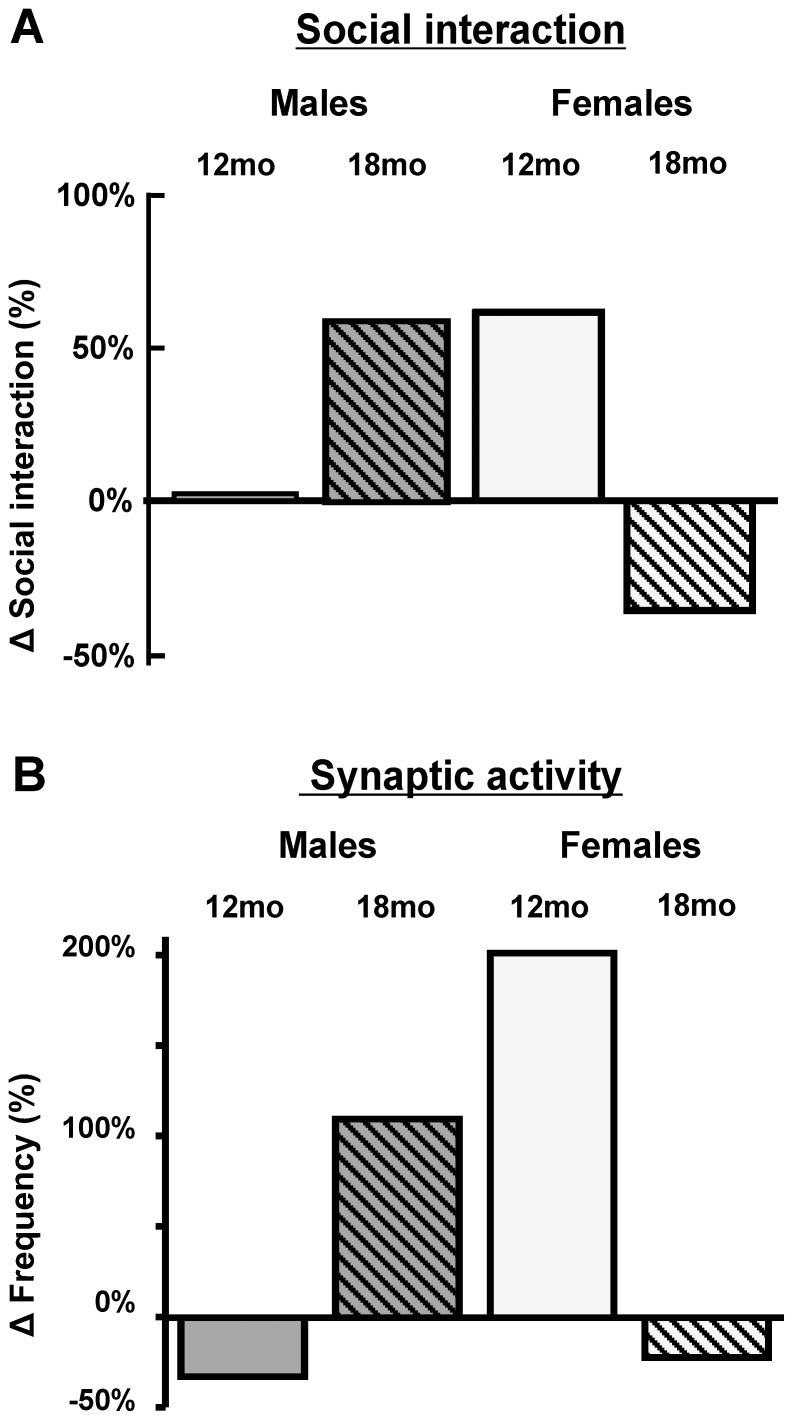
Age and sex-dependent variations in behaviour and synaptic activity in 3xTg-AD mice have the same signature. Illustration of sex-dependent changes in social interactions (A) and synaptic activity (B) in 12- and 18-month-old 3xTg mice when compared with NonTg mice, emphasizing the tight association between altered social interactions and changes in synaptic activity.

We demonstrated here that major age- and sex-dependent changes in social interactions can be observed in an animal model of AD, possibly reminiscent of neuropsychiatric symptoms of this prevalent disease. Our results are in line with recent studies reporting hyperactivity and social disabilities in transgenic mice of AD [13]–[15]. However, our study is the first, to our knowledge, to take into account both sex- and age-dependent differences in social behaviour. Furthermore the present study demonstrate that, while not matched with the expression of typical markers of AD neuropathology, this biphasic evolution of social interaction behaviour was associated with a variation in synaptic activity impinging on pyramidal cell in the medial prefrontal cortex, as summarized in Fig. 5 *A* and *B*.

The apparent uncoupling between typical AD biomarkers, such as Aβ and tau, and sex-dependent behavioural alterations corroborates previous evidence in the 3xTg-AD line [7]. On the other hand, the association between synaptic activity and social behaviour suggested by our results may be an important finding for future investigations. Indeed a reduction in neuronal activity in the mPFC has been shown to rely with social behaviour outcome, as observed in chronically defeated and apathic mice or clinically depressed patients [17], [18]. In contrast previous studies [19], [44] also demonstrated that an increase in medial prefrontal cortex activity through an enhancement in glutamate release or optogenetic-based stimulation of mPFC, can stimulate or restore normal social behaviour [17]–[19] of socially defeated mice. Altered background synaptic activity in cortical areas has been observed in several experimental models of AD (for review see [45]). Recent studies report that Aβ facilitates neuronal activity by potentiating neurotransmitter release in the hippocampus, which could thus lead to an aberrant network activity under normal circumstances [45]–[47] and may translate into memory deficits [45], [46].

In the present study, we observed a general increase in synaptic activity in the prefrontal cortex whereas the synaptic balance between excitatory and inhibitory currents within cortical cells remained unchanged. Such an increase in background synaptic activity is likely to disrupt frontal behaviour, through a decrease of signal–to-noise ratios and a consequent destabilization of neural networks [48]. For example, investigations using artificial networks indicate that a reduction of dopaminergic input into the prefrontal cortex alters the signal-to-noise ratio and results in less distinct cortical representation, as observed in AD or schizophrenic patients [49]–[52]. Taken together, these findings combined with our results suggest an association between altered background synaptic activity in mPFC and social dysfunction in AD.

Besides aging, the influence of sex was also critical, with females developing behavioural and electrophysiological abnormalities 6 months earlier than males. Sex differences in the development of AD have been suspected for some time, as several epidemiological studies report a higher incidence of AD in women [36], [53]. Data gathered on 3xTg-AD and Tg2576 mice provide histopathological evidence of increased Aβ deposits in females compared to males [54], [55]. Sexual dimorphism has also been observed in cognitive, mnemonic or stressful tasks in 3xTg-AD mice which, consistent with the present data, were not correlated with Aβ or tau pathologies [7], A similar sex-related mismatch between *post mortem* AD pathology and clinical manifestation of AD has been reported [35]. Interestingly, sexual dimorphism in background synaptic activity has been observed in fronto-subcortical areas [56], known to be involved in social behaviours and executive functions [38]. Such physiological differences could be amplified under pathological conditions, as observed in our study. Taken together, these findings argue that sex differences lay within the process of translation from neuronal pathology toward psychiatric-like symptoms.

Apathy and social disinhibition are observed in 76% and 30% of AD patients, respectively, and are two subtypes of personality changes which can be considered as opposite expression of a common behavioural domain, patients being either apathetic or disinhibited [2]–[4], [57]–[60]. Apathy is a particularly frequent and persistent neuropsychiatric symptom associated with AD and can be defined by a quantitative reduction in self-generated voluntary and purposeful behaviours [2], [4], [58]. It has been recently established that apathy is associated with an increase in risk of conversion from MCI to AD [61], [62] and with a faster overall cognitive decay [59]. In this context, and given the difficulty to treat the neuropsychiatric symptoms of AD, the present results combined with recent studies [13], [14], [63], [64] suggest notably that animal models can be instrumental to the development of new therapeutic interventions against apathy and disinhibition.

Our results represent, to our knowledge, the first demonstration that major sex-dependent changes in social behaviour can be observed at different ages in an animal model of AD. The identification of prefrontal cortex synaptic activity as a potential substrate of these behavioural defects provides a new window of opportunities for therapeutic intervention. The biphasic alterations in social responses we found in 3xTg-AD mice, a social disinhibition phase followed by apathy-like behaviour, suggest that 3xTg-AD mice could be used to investigate cellular substrates of some of the most complex AD-like behavioural symptoms consequent of frontal cortex dysfunction.

## Supporting Information

Table S1 (DOCX)

Table S2 (DOCX)

Table S3 (DOCX)
