# Supplementary material for: Sex-Dependent Alterations in Social Behaviour and Cortical Synaptic Activity Coincide at Different Ages in a Model of Alzheimer’s Disease
Source: PLoS One. 2012 Sep 24;7(9):e46111. doi: 10.1371/journal.pone.0046111 (PMC3454358; doi:10.1371/journal.pone.0046111)
Supplement: Table S1 — (DOCX) [file pone.0046111.s001.docx]

**Supplementary table 1: Details of social and non-social behavioural events assessed.**

|  | **Male** | | | | **Female** | | | |
| --- | --- | --- | --- | --- | --- | --- | --- | --- |
|  | 12 months | | 18 months | | 12 months | | 18 months | |
|  | NonTg | 3xTg-AD | NonTg | 3xTg-AD | NonTg | 3xTg-AD | NonTg | 3xTg-AD |
| **Social events** |  |  |  |  |  |  |  |  |
| Total number of events | 97.12 ± 6.51 | 99.37 ± 8.29 | 80.75 ± 7.8 | 127.62 ± 11.72****** | 55.5 ± 4.81 | 89.62 ± 9.19** | 78.37 ± 6.25 | 50 ± 8.43** |
| Sniffing | 76.25 ± 6.3 | 79.12 ± 8.34 | 62.37 ± 5.17 | 89.75 ± 6.92 | 48.37 ± 3.93 | 79.37 ± 7.2 | 62.12 ± 4.2 | 44.12 ± 6.72 |
| Grooming the partner | 9.75 ± 1.91 | 8.87 ± 1.98 | 6.37 ± 2.46 | 15.25 ± 4.2 | 3.75 ± 1.35 | 3.12 ± 0.95 | 5.87 ± 1.12 | 1.75 ± 0.75 |
| Crawling over/under | 7.0 ± 2.82 | 8.25 ± 2.75 | 10.37 ± 3.3 | 11.87 ± 3.81 | 2.5 ± 0.65 | 4.12 ± 1.06 | 8.75 ± 2.23 | 3.25 ± 1.38 |
| Following | 4.12 ± 1.66 | 3.12 ± 0.89 | 1.62 ± 0.68 | 10.75 ± 2.3 | 0.87 ± 0.61 | 3.0 ± 0.91 | 1.62 ± 0.73 | 0.87 ± 0.48 |
| **Non-social events** |  |  |  |  |  |  |  |  |
| Wrestling / biting | 0.12 ± 0.12 | 0 ± 0 | 0 ± 0 | 0 ± 0 | 0 ± 0 | 0 ± 0 | 0 ± 0 | 0 ± 0 |

Notes: ** p<0.01 Mann-Whitney test compared to age and sex matched NonTg animal.

Values are expressed as means ± SEM (n=8 animals per group)
