# Supplementary material for: Sex-Dependent Alterations in Social Behaviour and Cortical Synaptic Activity Coincide at Different Ages in a Model of Alzheimer’s Disease
Source: PLoS One. 2012 Sep 24;7(9):e46111. doi: 10.1371/journal.pone.0046111 (PMC3454358; doi:10.1371/journal.pone.0046111)
Supplement: Table S2 — (DOCX) [file pone.0046111.s002.docx]

Supplementary Table 2: Three-way ANOVA of mIPSC frequency

| **Source** | **SS** | **df** | **MS** | **F** | **P** |
| --- | --- | --- | --- | --- | --- |
| Age | 1.76 | 1 | 1.76 | 0.81 | 0.37 |
| Sex | 5.3 | 1 | 5.3 | 2.44 | 0.12 |
| Genotype | 9.74 | 1 | 9.74 | 4.49 | 0.039 |
| Age/Sex interaction | 1.91 | 1 | 1.91 | 0.88 | 0.35 |
| Age/Genotype | 0.24 | 1 | 0.24 | 0.11 | 0.74 |
| Sex/Genotype | 1.96 | 1 | 1.96 | 0.9 | 0.34 |
| Age/Sex/Genotype interaction | 18.34 | 1 | 18.34 | 8.45 | 0.005***** |
| Error | 99.75 | 46 | 2.17 |  |  |
| Total | 139 | 53 |  |  |  |
