# Supplementary material for: Sex-Dependent Alterations in Social Behaviour and Cortical Synaptic Activity Coincide at Different Ages in a Model of Alzheimer’s Disease
Source: PLoS One. 2012 Sep 24;7(9):e46111. doi: 10.1371/journal.pone.0046111 (PMC3454358; doi:10.1371/journal.pone.0046111)
Supplement: Table S3 — (DOCX) [file pone.0046111.s003.docx]

| **Source** | **SS** | **df** | **MS** | **F** | **P** |
| --- | --- | --- | --- | --- | --- |
| Age | 0.45 | 1 | 0.45 | 0.09 | 0.76 |
| Sex | 1.73 | 1 | 1.73 | 0.36 | 0.55 |
| Genotype | 0.71 | 1 | 0.71 | 0.15 | 0.7 |
| Age/Sex interaction | 2.93 | 1 | 2.93 | 0.61 | 0.43 |
| Age/Genotype | 2.19 | 1 | 2.19 | 0.46 | 0.5 |
| Sex/Genotype | 1.02 | 1 | 1.02 | 0.21 | 0.65 |
| Age/Sex/Genotype interaction | 39.55 | 1 | 39.55 | 8.27 | 0.006***** |
| Error | 191.01 | 40 | 4.78 |  |  |
| Total | 239.59 | 47 |  |  |  |

Supplementary Table 3: Three-way ANOVA of mEPSC frequency
